# Inflammatory Factors and Chronic Rhinosinusitis: An Umbrella Review

**DOI:** 10.7759/cureus.101782

**Published:** 2026-01-18

**Authors:** Stergios T Lialiaris, Konstantinos Chaidas, George Fyrmpas, Theodora Eleftheria Deftereou, Dimitra Spyroulia, Emmanuel P Prokopakis, Michael Katotomichelakis

**Affiliations:** 1 Εar, Nose, and Throat Department, University Hospital of Alexandroupolis, School of Medicine, Democritus University of Thrace, Alexandroupolis, GRC; 2 Histology-Embryology Department, University Hospital of Alexandroupolis, School of Medicine, Democritus University of Thrace, Alexandroupolis, GRC; 3 Department of Otolaryngology, "Korgialeneio – Benakeio" General Hospital of Athens, Athens, GRC; 4 Ear, Nose, and Throat Department, University General Hospital of Heraklion, Heraklion, GRC

**Keywords:** antibodies, cystic fibrosis, cytokines, monoclonal, nasal polyps, phenotype, rhinosinusitis

## Abstract

Chronic rhinosinusitis (CRS) is a common inflammatory disorder of the nasal mucosa and paranasal sinuses characterized by persistent sinonasal symptoms and objective endoscopic and imaging evidence of the disease. CRS is broadly described as a disease with two different phenotypes: without (CRSsNP) or with nasal polyps (CRSwNP), but substantial heterogeneity in clinical presentation and underlying mechanisms complicates classification and treatment selection. In accordance with the Preferred Reporting Items for Systematic Reviews and Meta-Analyses (PRISMA) guidelines, we conducted an umbrella review supported by a systematic literature search in Scopus and PubMed, screening publications from May 1968 to August 2025 and ultimately including 64 studies, with findings synthesized qualitatively. The evidence supported the concept that the inflammatory pathways in CRS reflect distinct, and sometimes overlapping, immune patterns dominated by T helper cells (Th1, Th2, Th17, Th22), and T regulatory cells (Treg). Type 1 inflammation, more commonly associated with CRSsNP, is characterized by interferon-gamma and interleukin (IL)-12 signaling and often shows prominent neutrophilic inflammation. Type 2 inflammation, particularly relevant to CRSwNP, involves epithelial-derived mediators such as thymic stromal lymphopoietin (TSLP), IL-25, and IL-33 and is defined by the presence of Th2 cytokines (IL-4, IL-5, and IL-13) and eosinophilic infiltration; downstream effects include increased mucus-related gene activity and changes in epithelial transport that may interact with other inflammatory programs. Type 3 (type 17) inflammation has been linked to increased IL-17 and IL-22 and is relevant to host defense against bacteria and fungi, including fungal rhinosinusitis and allergic fungal rhinosinusitis. Comorbid conditions, including aspirin-exacerbated respiratory disease and cystic fibrosis, further influence CRS endotypes and clinical severity. Biologic therapies, including dupilumab, highlight the potential of endotype-driven management, but additional work is needed to refine inflammatory classification, identify treatment-responsive subgroups, and reduce reliance on repeated surgery while mitigating progression to lower airway disease.

## Introduction and background

Chronic rhinosinusitis (CRS) is a serious health problem with a prevalence ranging from 5% to 12% [1]. CRS is described as inflammation of the nasal mucosa and/or the paranasal sinuses and the patients often complain about two or more of the following symptoms: nasal blockage, nasal obstruction, nasal congestion, or nasal discharge (anterior/posterior nasal drip); facial pain or pressure; and reduced sense of smell [1]. Rhinosinusitis can also lead to intracranial and ocular complications [2]. These symptoms should correlate with endoscopic signs and computed tomography (CT) findings [1]. To qualify as chronic, symptoms should persist for at least 12 weeks. Environmental and genetic factors, as well as factors associated with modern lifestyles (e.g., travel, environmental pollution, and diet), contribute to CRS and may help explain differences across populations [3].

CRS occurs either without (CRSsNP) or with nasal polyps (CRSwNP). It is heterogeneous in its clinical presentation and molecular pathophysiology, with variability in both phenotypes (observable clinical features) and endotypes (underlying biologic mechanisms). However, the specific mechanisms remain incompletely understood, which limits accurate classification [4,5].

Despite these differences, treatment options for CRS are typically focused on corticosteroids or sinonasal surgery. In 2019, dupilumab, a monoclonal antibody, was approved in the United States of America (USA) as a biologic therapy for CRSwNP. Dupilumab blocks interleukin IL-4 and/or IL-13 signaling by targeting the IL-4 receptor alpha chain. Other monoclonal antibodies, including those targeting the IL-5 receptor (benralizumab), immunoglobulin E (IgE; omalizumab), or IL-5 (mepolizumab), have been evaluated in clinical trials for polypoid CRS [6-8]. These newer therapies may enable more individualized treatment by matching therapy to specific CRS subtypes; however, available options remain limited, and further categorization is needed. Therefore, the objective of this umbrella review was to synthesize evidence on key inflammatory mediators and immune endotypes associated with CRS phenotypes (CRSsNP and CRSwNP) and to summarize how these pathways inform emerging endotype-driven management.

## Review

**Figure 1:**
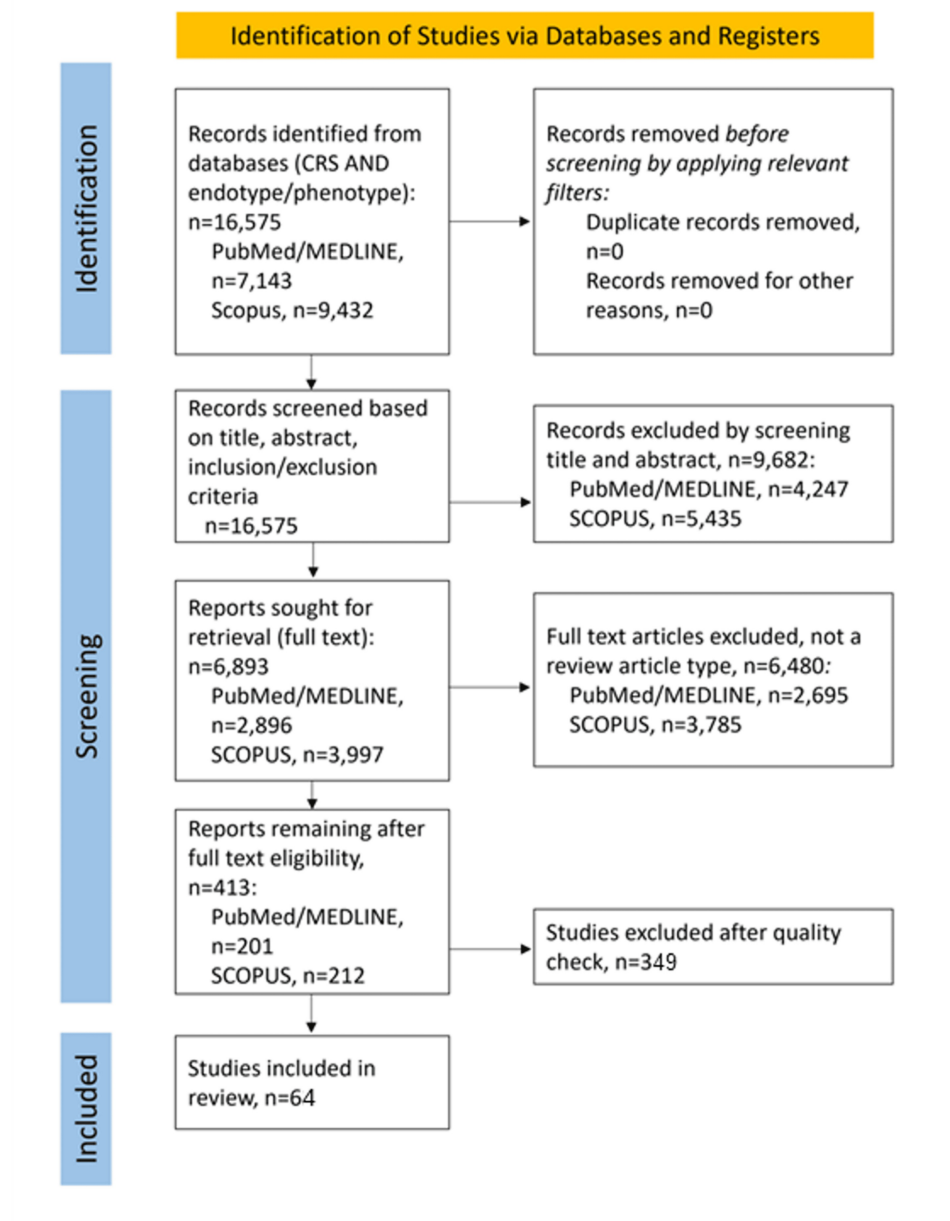
PRISMA flow diagram showing the selection of articles PRISMA: Preferred Reporting Items for Systematic Reviews and Meta-Analyses.

We conducted an umbrella review supported by a systematic literature search in two electronic databases (Scopus and PubMed) in accordance with the Preferred Reporting Items for Systematic Reviews and Meta-Analyses (PRISMA) guidelines [9]. Given the heterogeneity of evidence in this field, we synthesized findings qualitatively across multiple evidence types rather than performing a quantitative meta-analysis. We applied strict selection criteria, including English language, clear study protocols, and well-documented results. We searched the databases using the following keywords: “Chronic Rhinosinusitis,” “endotype,” “review,” and “phenotype.” We used keywords individually and in combination (e.g., “Chronic Rhinosinusitis” AND “endotype”) (Figure 1).

We initially screened the published literature from May 1968 to August 2025 that included an English-language abstract. We excluded non-human studies and studies published in languages other than English or without an English abstract. We included studies involving only adult populations. Based on these criteria, we ultimately included 64 studies/texts/works in this review. 

Results

**Figure 2:**
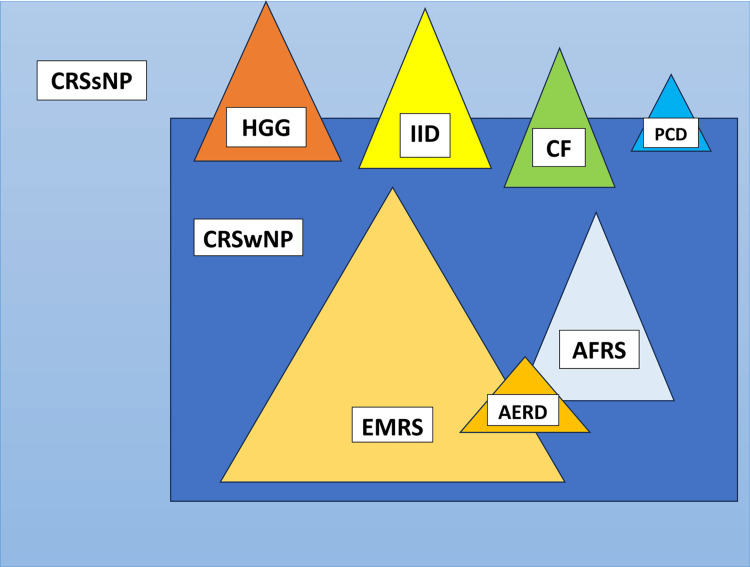
Various CRS phenotypes CRS, Chronic Rhinosinusitis; CRSsNP, Chronic Rhinosinusitis without nasal polyps; CRSwNP, Chronic Rhinosinusitis with nasal polyps; IID, Innate Immune defects; HGG, Hypogammaglobulinemia; CF, Cystic fibrosis; PCD, Primary ciliary dyskinesia; EMRS, Eosinophilic mucin rhinosinusitis; AFRS, Allergic fungal sinusitis; AERD, Aspirin-exacerbated respiratory disease. Image credit: Created by Lialiaris S using Microsoft Word (Microsoft Corp., Redmond, WA, USA).

To date, no widely accepted criteria guide the optimal therapeutic approach for CRSsNP or CRSwNP, and for many years, experts largely accepted the clinicopathological heterogeneity of CRS. Since 2004 [6], however, a group of rhinosinusitis experts has proposed that CRS can be divided into three main types: CRSwNP, CRSsNP, and allergic fungal rhinosinusitis (AFRS). Figure 2 shows the possible phenotypes of CRS, with CRSsNP comprising approximately 80% and CRSwNP approximately 20%; within CRSwNP, AFRS, eosinophilic mucin rhinosinusitis, and aspirin-exacerbated respiratory disease (AERD) exist as subsets [7-11].

Figure 2 also depicts the subsets of CRS associated with innate immune defects, hypogammaglobulinemia, cystic fibrosis (CF), and primary ciliary dyskinesia, which overlap both CRSwNP and CRSsNP with controversial rates. Other rare phenotypes are not listed in the diagram [7-11]. Nevertheless, CRS symptoms can vary substantially across patients.

Factors causing inflammation in CRS

**Figure 3:**
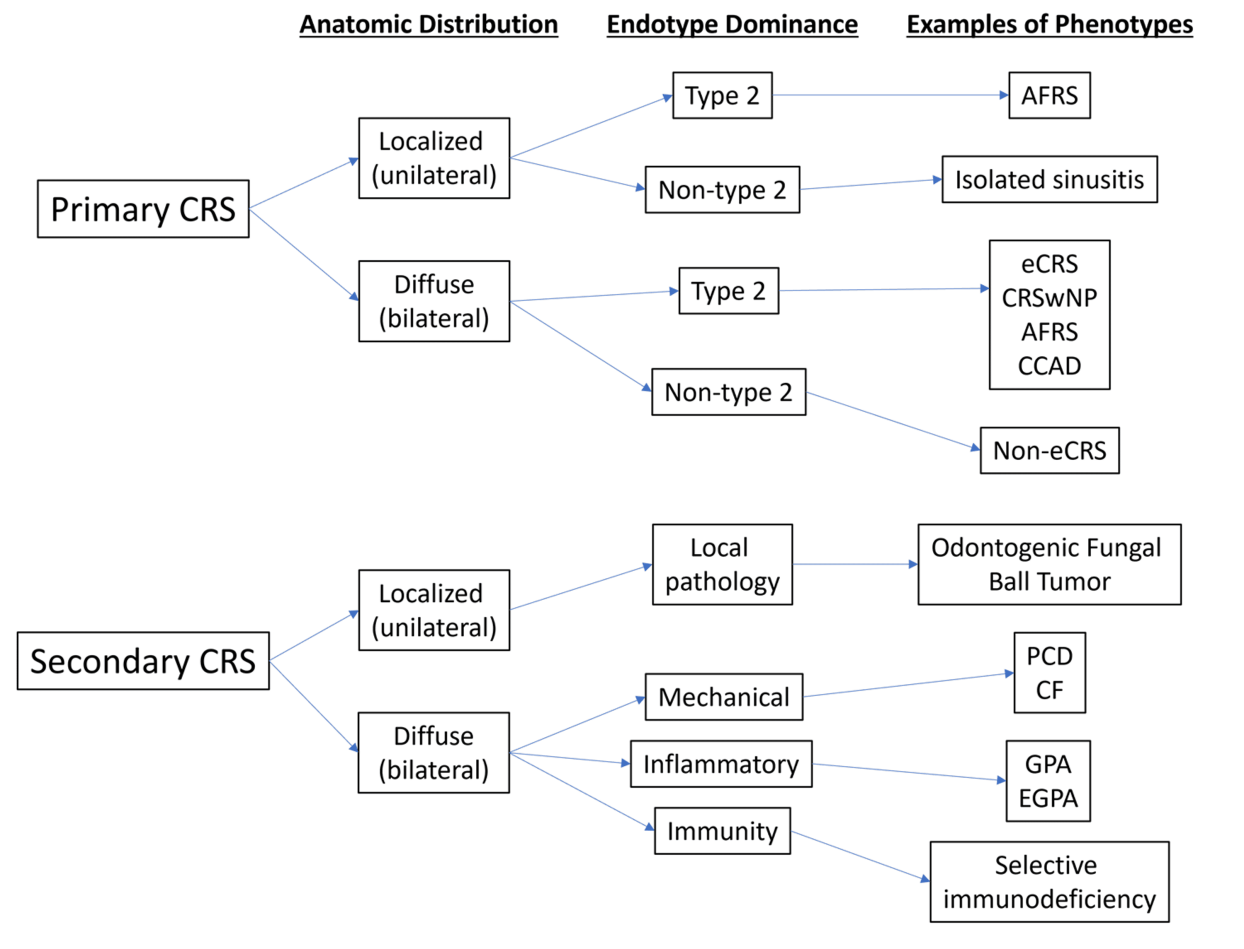
Classification of primary and secondary CRS AFRS, allergic fungal rhinosinusitis; CCAD, central compartment allergic disease; CRS, chronic rhinosinusitis; CRSwNP, chronic rhinosinusitis with nasal polyps; eCRS, eosinophilic CRS; OMC, ostiomeatal complex; PCD, primary ciliary diskinesia; CF, cystic fibrosis; GPA, granulomatosis with polyangiitis (Wegner’s disease); EGPA, eosinophilic granulomatosis with polyangiitis (Churg-Strauss disease) [1]. Image credit: Created by Lialiaris S using Microsoft Powerpoint (Microsoft Corp., Redmond, WA, USA).

To understand CRS in depth, the key pathophysiological mechanisms underlying its clinical manifestations must be clarified. Current concepts propose that inflammation of the nasal mucosa may be driven, in part, by epithelial barrier disruption and immune dysregulation. To fully explain the diversity of CRS, investigators must evaluate multiple inflammatory response patterns, and additional patterns may be identified in the future. Available evidence suggests that CRS inflammatory pathways are dominated by T helper cells (Th1, Th2, Th17, Th22), and regulatory T cells (Treg) (Figure 3) [1,12].

Investigators commonly describe three immune-inflammatory patterns that differ in the sequence of the inflammatory mediators, the immune cell populations involved, and the associated physiologic functions. Type 1 inflammation (often associated with CRSsNP) is associated with the presence of interferon-gamma (IFN-γ) and IL-12 produced by Th1 cells [13]. Type 2 inflammation (associated with CRS, particularly CRSwNP) is defined by producing IL-4, IL-5, and IL-13 by Th2 cells. Type 3 inflammation (also referred to as type 17 by some authors) is characterized by increased IL-17 and IL-22 produced by Th17 and Th22 cells, respectively [14-16]. In addition, Treg cell maturation promotes production of transforming growth factor beta (TGF-β) [17]. Table 1 describes the cytokines and other factors involved in CRS [1,5,18-40].

**Table 1:** Key inflammatory mediators implicated in CRS CRS, chronic rhinosinusitis; ECP, eosinophil cationic protein; IFN-γ, interferon gamma; HIV, human immunodeficiency virus; IgE, immunoglobulin E; IL, interleukin; MPO, myeloperoxidase; TNFα, tumor necrosis factor alpha; TGFβ, transforming growth factor beta.

Mediator	Family members	Year of discovery/first reports	Major source cells	Receptor(s)	Primary target cells/tissues	Key actions/functions	CRS inflammatory pattern	Year linked to CRS/CRS therapy	References
IL-1	IL-1α, IL-1β, IL-1Ra, IL-36α, IL-36β, IL-36γ, IL-36Ra, IL-18, IL-33, IL-37, IL-38	1943-48 / 1979	Monocytes, macrophages, B cells	IL1R1, IL1R2	Natural killer (NK) cells, Th cells, B cells, endothelial cells, other cells	Activation; stimulation; maturation; proliferation; inflammation	All types	2016	[5,18]
IL-2	IL-4, IL-7, IL-9, IL-15, IL-21	1943-48 / 1979	T helper 1 (Th1) cells	IL2RA, IL2RB, IL2RG	NK cells, T cells, B cells, macrophages	T-cell stimulation; treatment of cancer; transplantation; HIV	Type 1 and 3 inflammation	2012	[19]
IL-4	-	1982 / 1986	T helper 2 (Th2) cells, mast cells, macrophages, CD4+ T cells	IL4Rα	T cells, B cells, endothelial cells	Differentiation; proliferation; role in allergy	Type 2 inflammation	2016	[20]
IL-5	IL-5, IL-5Rα	1986	Th2 cells, mast cells, eosinophils	IL5Rα, IL5Rβ	B cells, eosinophils	Proliferation; differentiation	Type 2 inflammation	2006	[21,22]
IL-6	-	1986	B cells, Th2 cells, endothelial cells, macrophages	IL6Rα, IL6Rβ	T cells, B cells, hematopoietic cells	Differentiation; antibody secretion; inflammation	Type 1 and type 3 inflammation	2016	[5,23]
IL-8	-	1990	Macrophages, lymphocytes, epithelial cells, endothelial cells	IL8Rα, IL8Rβ	Neutrophils, basophils, lymphocytes	Neutrophil chemotaxis	Type 1 inflammation	2012	[24]
IL-9	-	1980s / 1990	Th2 cells, CD4+ T cells	IL9R	T cells, B cells	Potentiation; stimulation	Type 2 inflammation		[25]
IL-10	-	1995	B cells, Th2 cells, CD8+ T cells, mast cells, macrophages	IL10Rα, IL10Rβ	B cells, Th1/Th2 cells, mast cells, macrophages	Activation; stimulation; inhibition of IFN and TNF production	Type 1 inflammation	2000	[26]
IL-12	-	1998	B cells, T cells, macrophages	IL12Rβ1, IL12Rβ2	NK cells, T cells	Differentiation; stimulation of IL-2, IFN-γ, TNF-α, and IL-10	Type 1 inflammation	2007	[27]
IL-13	-	1993	NK cells, Th2 cells, mast cells	IL13R	B cells, Th2 cells, macrophages	Stimulation; differentiation; inhibition of cytokine production (e.g., IL-1, IL-6, IL-8, IL-10, IL-12)	Type 2 inflammation	2016	[20,28]
IL-17	-	2002	T helper 17 (Th17) cells	IL17Rα, IL17Rβ	Endothelial cells, epithelial cells	Angiogenesis; inflammation	Type 3 inflammation	2001	[29,30]
IL-22	-	2000	Th17 cells	IL22R	-	Production; activation	Type 3 inflammation	2016	[5, 31]
IL-25	-	2001	Mast cells, eosinophils, macrophages, epithelial cells, T cells	LY6Ε	-	Induces IL-4, IL-5, and IL-13	Type 2 and 3 inflammation	2010	[32]
IL-33	-	2010	Multiple cell types (e.g., macrophages, endothelial cells, epithelial cells)	-	-	Induces T-helper cells to produce type 2 cytokines	Type 2 inflammation	2014	[33]
TNFα	-	1968	Macrophages	TNFR1, TNFR2	Immune system	Adipokine: promotes insulin resistance; cytokine: mediates cell signaling	Type 1 and non–type 2 inflammation	2016	[5,34]
TGFβ	-	1997	All white blood cell lineages, including macrophages	TGF-β1 to 3	-	Regulates key cellular activities	Type 1, non–type 2, and type 3 inflammation	2016	[5,35]
IFN-γ	-	1965	Adaptive immune cells	IFNGR1, IFNGR2	NK cells, CD4+ Th1 cells, CD8+ cytotoxic T cells	Type II interferon; regulates target-cell immune responses and shapes signaling within the immune system	Type 1, 2, and 3 inflammation	2016	[5,36]
ECP	-	1989	Eosinophil primary matrix	-	-	Member of the ribonuclease A superfamily; neurotoxic, helminthotoxic, and ribonucleolytic activities	Type 1, 2, and 3 inflammation	1997	[37]
MPO	-	1983	Neutrophils	-	-	Catalyzes the conversion of hydrogen peroxide and chloride ions into hypochlorous acid	Type 1 and non–type 2 inflammation	2005	[38]
IgE	-	1966	Plasma cells	FcεRI (type I Fcε receptor), FcεRII (type II Fcε receptor)	-	Antibody in mammals	Type 3 inflammation	2005	[39]

Type 1 inflammation

The literature often describes type 1 inflammation as non-type 2 inflammation, and CRS most commonly presents without nasal polyps. This pattern also shows features of mixed type 1 and type 3 inflammation with prominent neutrophil infiltration. Pathogen invasion of the nasal epithelium triggers epithelial release of IL-6, IL-8, tumor necrosis factor alpha, and other chemokines. Pattern-associated molecular pattern/Toll-like receptor immune responses appear to stimulate production of IFN-γ and IL-8 [40,41]. These responses promote IL-8 release and recruit immune cells (e.g., neutrophils) to the paranasal sinuses, thereby shaping downstream immune responses. Neutrophils subsequently release mediators including IL-1, IL-6, IL-8, and myeloperoxidase, an enzyme derived from neutrophil granules [42]. In response to pathogens, epithelial IFN-γ promotes differentiation of CD4+ T cells into Th1 cells [40,41]. Epithelial IL-6 promotes differentiation of CD4+ T cells into Th17 and Th22 cells; Th17 cells secrete IL-17 and IL-22, whereas Th22 cells secrete IL-22 [16].

Studies have reported upregulation of Treg cells in patients with CRSsNP compared with normal individuals, whereas patients with CRSwNP show downregulation [43]. In immune regulation, Treg cells downregulate Th1 and Th2 activity and influence IL-10 production [44]. Evidence indicates that TGF, a cytokine family member that may contribute to tissue growth in CRSsNP, is produced by Treg cells [44]. In addition, TGF-β plays a key role in fibroblast growth. Conversely, increased extracellular matrix synthesis, which contributes to upper airway epithelial remodeling, may increase symptom burden in CRSsNP [45]. TGF-β also participates in the differentiation of CD4+ T cells toward Th17 cells and in the maturation of Treg cells [43,45]. The precise role of Treg cells in CRS, including their capacity to reduce inflammation via IL-10 production and their potential contribution to airway remodeling and fibrosis, remain under investigation.

Type 2 inflammation

Type 2 inflammation in CRS has been well described. Nasal epithelial cells can be stimulated to secrete thymic stromal lymphopoietin (TSLP) [46,47], IL-25, and IL-33, which promote production of IL-4, IL-5, and IL-13 from epithelial cells and mucosal mast cells [48,49]. In addition, TSLP and IL-13 induce type 2 cytokine production by innate lymphoid cells [50]. Evidence also suggests that TSLP stimulates myeloid dendritic cells (mDCs) [49,51]. Once activated, mDCs present antigen and provide costimulatory signals that promote CD4+ T-cell differentiation toward Th2 cells. Th2 cells produce type 2 cytokines, which are central to type 2 inflammation. In an IL-4-rich environment that promotes Th2 expansion (Table 1), IL-5 supports eosinophil infiltration, which can induce eosinophilic extracellular traps, release of toxic proteins, and inflammation [52]. IL-4 and IL-13 upregulate mucin 5AC and mucin 5B gene activity, which encodes mucins and increases pendrin production [53]. Pendrin, an epithelial anion transporter, increases mucus production [54] and may enhance type 3 (or type 17) inflammation, with increased IL-17 and IL-22 levels [14,15] and by causing hypoxia [55].

Type 3 inflammation: fungal rhinosinusitis

Fungal diseases of the nose and paranasal sinuses are collectively termed fungal rhinosinusitis (FR). FR is broadly classified as invasive or noninvasive. Invasive FR involves fungal hyphae infiltrating the mucosa of the nose and paranasal sinuses [56]. Acute, chronic, and granulomatous invasive FR have been described [57]. Immunocompromised patients, including those with uncontrolled diabetes, are prone to invasive FR. Poor survival has been reported in patients of advanced age, those with intracranial extension, and those who do not undergo surgery as part of the treatment protocol [58].

AFRS is characterized by Th2 inflammation and fungal colonization of the nasal mucosa. Dematiaceous fungi and Aspergillus species account for most cases [57,59]. In AFRS, allergic inflammation occurs without fungal tissue invasion. AFRS may be unilateral or bilateral and can produce “allergic mucus” or “eosinophilic mucus,” which are thick inflammatory secretions characterized by tenacious mucus containing clusters of eosinophils and free eosinophilic cocci [60]. Another noninvasive form is the fungal ball, which typically occurs in middle-aged or older patients and presents as unilateral sinusitis, most often involving the maxillary sinuses, with microcalcification patterns on CT [61]. Fungal ball disease is often asymptomatic and may be detected incidentally on CT or magnetic resonance imaging (MRI).

Aspirin-exacerbated respiratory disease (AERD) and cystic fibrosis (CF)

AERD is a CRS syndrome characterized by eosinophilic nasal polyps, asthma, and respiratory reactions induced by aspirin and other nonsteroidal anti-inflammatory drugs (NSAIDs) that inhibit cyclooxygenase-1, with onset typically in adulthood [62]. Its prevalence is approximately 30% in patients with asthma and CRSwNP [63]. Respiratory reactions to aspirin and other NSAIDs are common in patients with CF, and mutations in the CF transmembrane conductance regulator gene are also reported in non-CF patients with CRS [64]. Across all ages, patients with CF have an increased prevalence of CRS. Management of CRS in CF is challenging due to increased mucus viscosity, chronic sinus infections, bacterial colonization and biofilm formation, and the presence of distinct sinus pathogens.

Discussion

CRS is a major focus in otorhinolaryngology, and research in this field continues to expand. The number of patients affected by CRS appears to be increasing, and investigators continue to evaluate mechanisms and therapies to improve outcomes [4,5]. The defining feature of CRS is persistent inflammation of the nasal mucosa, which may occur with or without nasal polyps. CRS is heterogeneous in both clinical presentation and underlying pathophysiology, including variability in phenotypes and genotypes. However, pathogenic mechanisms remain incompletely understood, limiting precise classification [4,5].

Our review of the international literature suggests that Th1 responses are characterized by IFN-γ and IL-12 expression, whereas Th2 responses are associated with elevated IL-4, IL-5, and IL-13 levels. More recently, type 3 (or type 17) inflammation has been described and is associated with increased IL-17 and IL-22 production [14,15]. Th1 responses primarily protect against intracellular microbes, including viruses. In contrast, type 2 inflammation contributes to immune responses against parasitic infections and is also linked to allergic rhinosinusitis. Type 3 inflammation is thought to support host defense against bacteria and fungi [14,15,19-23].

Geographic variation, age, asthma, fungi, and other infectious agents or medications may influence inflammatory pathways in CRS. Accordingly, CRS inflammation can show mixed type 1, type 2, and type 3 profiles across classic phenotypes with or without nasal polyps [1-5]. Endotype-based evaluation may help distinguish biologically distinct forms of CRS and support the selection of more individualized treatment strategies. Defining each patient’s inflammatory pattern may facilitate targeted therapy, and several biologic therapies are already available for selected patients with CRS. Continued research is needed to refine CRS phenotypes and endotypes and to advance individualized treatment approaches.

Limitations

This umbrella review has several limitations. First, the search strategy was limited to two electronic databases (Scopus and PubMed) and a predefined set of keywords, potentially missing relevant studies indexed in other databases or described using different terminology. Second, we restricted inclusion to studies published in English or with an English abstract and to adult populations, which may limit generalizability and introduce language- and selection-related bias. Third, the review spans literature published between May 1968 and August 2025. During this period, definitions, diagnostic criteria, endoscopic scoring systems, imaging practices, and molecular characterization methods for CRS have evolved substantially, thereby increasing heterogeneity and reducing comparability across included studies. Fourth, the included body of evidence likely reflects variability in how phenotypes and endotypes were defined and measured, including differences in tissue sampling sites, laboratory platforms, cytokine panels, and thresholds used to classify inflammatory patterns, which may limit synthesis into a unified framework. Given these limitations, we added a risk-of-bias table (Table 2) to provide a structured appraisal of study quality; however, we did not quantitatively pool findings across studies.

**Table 2:** Risk-of-bias assessment table of included studies Risk-of-bias was assessed according to study design. Narrative reviews were evaluated using the SANRA tool, clinical practice guidelines using AGREE II, systematic reviews using AMSTAR-2, and observational studies using the Newcastle–Ottawa Scale. The other references in the review [13,16-20,24-27,31-34,36,37,48,49] are basic science/ immunology/mechanistic studies (cell biology, cytokines, molecular pathways, animal studies), for which a traditional clinical Risk-of-Bias table does not apply. AGREE II, Appraisal of Guidelines for Research and Evaluation II; AMSTAR-2, A Measurement Tool to Assess Systematic Reviews 2; NOS, Newcastle–Ottawa Scale; SANRA, Scale for the Assessment of Narrative Review Articles.

Study	Study Design	Risk of Bias	Score	Tool	Main Domains Assessed	Overall Risk
Fokkens et al., 2020 (EPOS 2020) [1]	Clinical practice guideline	Assessed	AGREE II (domain-based)	AGREE II	Scope & purpose; Stakeholder involvement; Rigor; Clarity; Applicability; Editorial independence	Low
Payne et al., 2025 [2]	Clinical practice guideline	Assessed	AGREE II (domain-based)	AGREE II	Scope; Rigor; Clarity; Applicability; Editorial independence	Low–Moderate
Mattos et al., 2019 [3]	Observational study	Assessed	NOS 7–8/9	Newcastle–Ottawa Scale	Selection; Comparability; Outcome	Low
Van Crombruggen et al., 2011 [4]	Narrative review	Assessed	SANRA 10/12	SANRA	Justification; Literature search; Referencing; Scientific reasoning	Low
Tomassen et al., 2016 [5]	Observational study	Assessed	NOS 7–8/9	Newcastle–Ottawa Scale	Selection; Comparability; Outcome	Low
Meltzer et al., 2004 [6]	Consensus review	Assessed	SANRA 11/12	SANRA	Justification; Literature search; Referencing; Scientific reasoning	Low
Cho et al., 2020 [7]	Narrative review	Assessed	SANRA 10/12	SANRA	Justification; Literature search; Referencing; Scientific reasoning	Low
Avdeeva & Fokkens, 2018 [8]	Narrative review	Assessed	SANRA 9/12	SANRA	Justification; Literature search; Referencing; Scientific reasoning	Moderate
Papacharalampous et al., 2024 [9]	Systematic review	Assessed	AMSTAR-2: Moderate	AMSTAR-2	Protocol; Study selection; Risk of bias; Synthesis	Moderate
Staudacher et al., 2020 [10]	Narrative review	Assessed	SANRA 9/12	SANRA	Justification; Literature search; Referencing; Scientific reasoning	Moderate
Ahern & Cervin, 2019 [11]	Narrative review	Assessed	SANRA 9/12	SANRA	Justification; Literature search; Referencing; Scientific reasoning	Moderate
Wang et al., 2016 [12]	Observational study	Assessed	NOS 7–8/9	Newcastle–Ottawa Scale	Selection; Comparability; Outcome	Low
Schleimer, 2017 [14]	Narrative review	Assessed	SANRA 11/12	SANRA	Justification; Literature search; Referencing; Scientific reasoning	Low
Cao et al., 2019 [15]	Narrative review	Assessed	SANRA 10/12	SANRA	Justification; Literature search; Referencing; Scientific reasoning	Low
Bachert et al., 1997 [21]	Observational study	Assessed	NOS 7–8/9	Newcastle–Ottawa Scale	Selection; Comparability; Outcome	Low
Gevaert et al., 2006 [22]	Observational study	Assessed	NOS 7–8/9	Newcastle–Ottawa Scale	Selection; Comparability; Outcome	Low
Hirano et al., 1986 [23]	Observational study	Assessed	NOS 7–8/9	Newcastle–Ottawa Scale	Selection; Comparability; Outcome	Low
Seyfizadeh et al., 2015 [28]	Narrative review	Assessed	SANRA 8/12	SANRA	Justification; Literature search; Referencing; Scientific reasoning	Moderate
Aggarwal & Gurney, 2002 [29]	Narrative review	Assessed	SANRA 8/12	SANRA	Justification; Literature search; Referencing; Scientific reasoning	Moderate
Tesmer et al., 2008 [30]	Narrative review	Assessed	SANRA 9/12	SANRA	Justification; Literature search; Referencing; Scientific reasoning	Moderate
Massagué, 2012 [35]	Narrative review	Assessed	SANRA 11/12	SANRA	Justification; Literature search; Referencing; Scientific reasoning	Low
Klebanoff, 2005 [38]	Narrative review	Assessed	SANRA 10/12	SANRA	Justification; Literature search; Referencing; Scientific reasoning	Low
Takhar et al., 2005 [39]	Observational study	Assessed	NOS 7–8/9	Newcastle–Ottawa Scale	Selection; Comparability; Outcome	Low
Akdis et al., 2013 [40]	Consensus review	Assessed	SANRA 11/12	SANRA	Justification; Literature search; Referencing; Scientific reasoning	Low
Hirschberg et al., 2016 [41]	Observational study	Assessed	NOS 7–8/9	Newcastle–Ottawa Scale	Selection; Comparability; Outcome	Low
Bachert et al., 2001 [42]	Observational study	Assessed	NOS 7–8/9	Newcastle–Ottawa Scale	Selection; Comparability; Outcome	Low
Van Bruaene et al., 2008 [43]	Observational study	Assessed	NOS 7–8/9	Newcastle–Ottawa Scale	Selection; Comparability; Outcome	Low
Gratz et al., 2013 [44]	Narrative review	Assessed	SANRA 9/12	SANRA	Justification; Literature search; Referencing; Scientific reasoning	Moderate
Van Bruaene et al., 2009 [45]	Observational study	Assessed	NOS 7–8/9	Newcastle–Ottawa Scale	Selection; Comparability; Outcome	Low
Al-Alawi et al., 2014 [46]	Narrative review	Assessed	SANRA 9/12	SANRA	Justification; Literature search; Referencing; Scientific reasoning	Moderate
Licona-Limon et al., 2013 [47]	Narrative review	Assessed	SANRA 10/12	SANRA	Justification; Literature search; Referencing; Scientific reasoning	Low
Shaw et al., 2013 [50]	Observational study	Assessed	NOS 7–8/9	Newcastle–Ottawa Scale	Selection; Comparability; Outcome	Low
Kato et al., 2007 [51]	Observational study	Assessed	NOS 7–8/9	Newcastle–Ottawa Scale	Selection; Comparability; Outcome	Low
Hamilos et al., 1995 [52]	Observational study	Assessed	NOS 7–8/9	Newcastle–Ottawa Scale	Selection; Comparability; Outcome	Low
Zhang et al., 2018 [53]	Observational study	Assessed	NOS 7–8/9	Newcastle–Ottawa Scale	Selection; Comparability; Outcome	Low
Seshadri et al., 2015 [54]	Observational study	Assessed	NOS 7–8/9	Newcastle–Ottawa Scale	Selection; Comparability; Outcome	Low
Cho & Kim, 2018 [55]	Narrative review	Assessed	SANRA 9/12	SANRA	Justification; Literature search; Referencing; Scientific reasoning	Moderate
Ni Mhurchu et al., 2017 [56]	Observational study	Assessed	NOS 7–8/9	Newcastle–Ottawa Scale	Selection; Comparability; Outcome	Low
Montone et al., 2012 [57]	Observational study	Assessed	NOS 7–8/9	Newcastle–Ottawa Scale	Selection; Comparability; Outcome	Low
Turner et al., 2013 [58]	Systematic review	Assessed	AMSTAR-2: Moderate	AMSTAR-2	Search strategy; Selection; Outcome synthesis	Moderate
Steinke & Borish, 2016 [59]	Narrative review	Assessed	SANRA 9/12	SANRA	Justification; Literature search; Referencing; Scientific reasoning	Moderate
Aeumjaturapat et al., 2003 [60]	Observational study	Assessed	NOS 7–8/9	Newcastle–Ottawa Scale	Selection; Comparability; Outcome	Low
Dhong et al., 2000 [61]	Observational study	Assessed	NOS 7–8/9	Newcastle–Ottawa Scale	Selection; Comparability; Outcome	Low
Szczeklik et al., 2000 [62]	Observational study	Assessed	NOS 7–8/9	Newcastle–Ottawa Scale	Selection; Comparability; Outcome	Low
Rajan et al., 2015 [63]	Meta-analysis	Assessed	AMSTAR-2: Moderate	AMSTAR-2	Study selection; Bias assessment; Statistical synthesis	Moderate
Farrell, 2008 [64]	Observational study	Assessed	NOS 7–8/9	Newcastle–Ottawa Scale	Selection; Comparability; Outcome	Low

Finally, publication bias and selective reporting cannot be excluded, particularly for emerging biologic targets or therapies.

## Conclusions

Multiple studies have examined CRS phenotypes and endotypes. Further progress is expected as technological advances improve the identification of CRS subtypes. In parallel, ongoing investigation of biologic factors, including thymic stromal lymphopoietin, IL-33, and IL-25, may further distinguish known CRS endotypes and help identify patients who do or do not respond to specific agents. Personalized management of upper airway disease, including CRSwNP, should be a priority in the coming decade, particularly for patients with severe CRS. Biologic therapies may offer additional treatment options once patients are categorized by phenotype and endotype. However, broader implementation requires improved understanding of which monoclonal antibody is most appropriate for each CRS endotype and phenotype. Earlier identification of these subgroups at disease onset may support earlier treatment, reduce the need for repeated surgical procedures in subsequent years, and potentially lower the risk of progression to lower airway disease.
